# Agonist of PPAR-γ Reduced Epithelial-Mesenchymal Transition in Eosinophilic Chronic Rhinosinusitis with Nasal Polyps via Inhibition of High Mobility Group Box1

**DOI:** 10.7150/ijms.35936

**Published:** 2019-11-09

**Authors:** Pingli Yang, Shan Chen, Gang Zhong, Weijia Kong, Yanjun Wang

**Affiliations:** 1 Department of otorhinolaryngology, Union Hospital, Tongji Medical College, Huazhong University of Science and Technology, Wuhan, 430022, China; 2 Institutes of Otorhinolaryngology, Union Hospital, Tongji Medical College, Huazhong University of Science and Technology, Wuhan, 430022, China; 3 Department of otorhinolaryngology, The First Affiliated Hospital, Shihezi University School of Medicine, Shihezi, Xinjiang, 832000, China

**Keywords:** Epithelial-mesenchymal transition, chronic rhinosinusitis with nasal polyps, high mobility group box 1, peroxisome proliferator-activated receptor-γ

## Abstract

Epithelial-mesenchymal transition (EMT) has been reported to occur in eosinophilic chronic rhinosinusitis with nasal polyps (ECRSwNP). Among the cytokines that cause EMT, high mobility group box 1 (HMGB1) has been shown to give rise to EMT in airway epithelial cells. However, the mechanism of HMGB1-induced EMT in ECRSwNP is unknown. We explored the mechanism and possible inhibitor. Immunohistochemistry (IHC), immunofluorescence (IF), and western blot assay were used to detect the expression and location of HMGB1, peroxisome proliferator-activated receptor-γ (PPAR-γ), and EMT markers in eighteen ECRSwNP and twelve normal nasal mucosa tissues. Epithelial cells isolated from ECRSwNP were cultured with various doses of recombinant human HMGB1 (rhHMGB1) to study the expression of PPAR-γ, and EMT markers. Additionally, the ligand of PPAR-γ was incubated with epithelial cells to interfere with the effects of lipopolysaccharide (LPS) or rhHMGB1 to explore the effect on expression of HMGB1 and EMT markers. These results suggest that HMGB1 was highly expressed in ECRSwNP compared with its expression in control tissues, and EMT was also found highly in ECRSwNP compared with control tissues. Moreover, the cytoplasmic accumulation of HMGB1 in ECRSwNP was obvious compared with normal tissues. We also found dose-dependent induction by rhHMGB1 of up-regulation of N-cadherin and vimentin and down-regulation of ZO-1 and E-cadherin in epithelial cells isolated from ECRSwNP. The agonist of PPAR-γ not only reduced release of HMGB1 induced by LPS, but also reversed the EMT. The protective role of PPAR-γ also appeared in cells that had been incubated with rhHMGB1. In the current study, we discovered that the agonist of PPAR-γ has a potential role in inhibited HMGB1-induced EMT in ECRSwNP. The agonist of PPAR-γ may contribute to inhabit epithelial cells to become mesenchymal-like cells which play an important role in the pathogenesis of ECRSwNP.

## Introduction

Chronic rhinosinusitis with nasal polyps (CRSwNP) is characterized by an intensely edematous stroma with formation of pseudocysts, infiltration of subepithelial and perivascular inflammatory cells, and albumin deposition 1. Phenotyping classification of CRSwNP was inappropriate nowadays because it is not adequately reflect the pathophysiologic diversity, while endotype, based on the pathogenesis is more helpful for the diagnosis and treatment for CRSwNP 2. Endotype, which was defined by different pathogenic mechanisms, currently being accepted by many authors for it is correlate with the disease's clinical manifestations and behavior 3-5. Based on endotype, regardless of racial backgrounds, geographic areas and populations, CRSwNP can be divided into eosinophilic (ECRSwNP), when the nasal mucosa shows considerable eosinophilic infiltration, and non-eosinophilic (nonECRSwNP), when it does not 6-9. The two types of CRSwNP show different patterns of inflammation and tissue remodeling. About the changes in inflammation patterns, ECRSwNP show eosinophilic inflammation with typical type 2-biased immune response, whereas nonECRSwNP demonstrates type 1/type 3 predominant immune responses and it also shows a few eosinophils compared with CRSsNP 10. Type 2-biased immune responses are featured by upregulated production of IL-4, IL-5, IL-13 and local IgE 11. IL-5 regulates the activity of eosinophils and there are positive interactions between them 12. As regard to tissue remodeling, basement membrane was thicker and squamous metaplasia of epithelium was more frequently in eosinophilic inflammation 13. In previous studies, HMGB1 has been shown to support the survival of eosinophilic granulocytes 14 and has been confirmed to relate to tissue remodeling, such as in COPD 15,16. Extracellular HMGB1 amplifies inflammatory and remodeling signals through binding to RAGE or other possibly partners into target cells while intranuclear HMGB1 could be released to target cells too. Luisa et al. found that HMGB1 translocated from nuclei to cytoplasm and observed a positive relationship between HMGB1 and eosinophil scores after stimulating with lipopolysaccharide (LPS) in human nasal epithelial cells, suggesting that HMGB1 plays an important role in ECRSwNP 17, but the mechanism of the relationship between HMGB1 and formation of nasal polyp in ECRSwNP is unclear.

HMGB1 is a pro-inflammatory DNA-binding nuclear protein. It can initiate and promote inflammatory cytokine synthesis by forming complexes with pro-inflammatory molecules when actively secreted by immune cells after exposure to a danger signal or when passively released by dead or dying cells 15,18. Previous studies showed that HMGB1 induces epithelial-mesenchymal transition (EMT) in airway epithelial tissue 19,20. EMT, the transition of epithelial and endothelial cells to a motile phenotype, namely, transformation to mesenchymal stem cells, which not only associated with embryonic development, inflammation, tissue regeneration, and tumor formation, but also play an important role in the pathogenesis of ECRSwNP 21-23. At the molecular level, epithelial cells lose their epithelial character and gain mesenchymal properties. Associated changes are the down-regulation of epithelial markers such as ZO-1 and E-cadherin, and the up-regulation of mesenchymal markers such as vimentin and N-cadherin 24. We speculate that HMGB1 and eosinophils promote each other and cause the acceleration of the EMT process, thus playing an important role in the pathogenesis of ECRSwNP.

Peroxisome proliferator-activated receptor-γ (PPAR-γ) is a member of a class of PPARs that can be activated by specific PPAR-γ ligands in the cytoplasm. Numerous studies have demonstrated that PPAR-γ activated by ligands can suppress such biological functions of HMGB1 as extracellular release, gene expression, and signaling pathways 25-27. PPAR-γ ligands contain fibrates, thiazolidinediones, and eicosanoids 28. Rosiglitazone (ROG), an antidiabetic drug belonging to the thiazolidinedione class, has been reported to be involved in the inhibition of LPS-induced HMGB1 release in RAW264.7 cells via activated PPAR-γ 29. Therefore, we speculated that the agonist of PPAR-γ may reduce HMGB1-induced EMT in ECRSwNP.

## Materials and Methods

### Patient tissue samples

Eighteen ECRSwNP tissue samples named experimental group and twelve normal nasal mucosa tissue samples named control were obtained from the Department of Otolaryngology, Union Hospital (Wuhan, China) between January and December 2018. ECRSwNP samples were classified as such when the number of eosinophils counted at high-power field (HPF) (×400) exceeded 10, after 10 high-power fields were randomly selected and analyzed. Tissues of ECRSwNP were diagnosed as nasal polyps by the Department of Pathology at Union Hospital, whereas normal nasal mucosa tissues were obtained from patients who required partial excision of the turbinate, uncinate process and ethmoidal mucosa for surgical purposes, for example deviated nasal septum, or intracranial access. Each tissue was divided into three parts, one of it was reserved for cell culture, one of it was fixed for immunohistochemistry (IHC) and immunofluorescence (IF) evaluation using formalin for paraffin section or frozen section, and the last one stored at -80°C for protein extraction. We obtained informed consent from each patient and the study protocol was approved by the research ethics committee of Tongji Medical College, Huazhong University of Science and Technology (Permit Number: S440).

### Isolation of primary human nasal epithelial cells

Tissue samples of ECRSwNP of approximately 1 ml volume were rinsed with normal saline, transferred into 10 ml of DMEM/F12 medium containing 1% penicillin/streptomycin (Hyclone, USA), and digested with 0.1% protease from Streptomyces griseus (9036-06-0, CAS, USA) and 0.1 mg/ml deoxyribonuclease (D5025, Sigma, USA), and incubated at 4°C overnight. Epithelial cells were removed by gently scraping and blow them into single cell to the greatest extent. The medium was then transferred into a 15-ml conical tube, centrifuged at 1500 rpm for 5 min, the supernatant decanted, and the pellet resuspended in DMEM/F12 medium. Primary cells were plated on collagen type IV (Sigma)-coated 12-well culture plates with density of 2×10^5^cells/well for protein extraction and on 24-well culture plates with density of 1×10^5^cells/well for IF, and then cultured at 37°C in 5% CO2 overnight. The medium was then changed to PneumaCultTM-EX plus Basal Medium (05041, CAT, USA) containing 1% penicillin/streptomycin. For EMT induction, recombinant human HMGB1 (P09429, Bio-techne, USA) was added into the plates at different doses (100, 300, 500 ng/ml) for 48 h. Tissues derived from multiple patients and stimulation experiments consisted of a minimum of three independent experiments.

### Immunohistochemical staining

The tissues were fixed with phosphate buffered saline (PBS) containing 4% formaldehyde for 24 h (4°C, pH 7.3), dehydrated in a graded ethanol series (70%, 80%, 90%, 95%, and 100%), embedded in paraffin, and then sliced into sections of 5-μm thickness and placed on poly-L-lysine-coated glass slides.

The paraffin glass slides of ECRSwNP and control for IHC were deparaffinized and hydrated in a graded ethanol series (100%, 95%, 90%, 80%, and 70%), subjected to heat-induced epitope retrieval, washed three times with PBS, and then incubated with 3% H_2_O_2_ for 10 min to block endogenous peroxidases. Bovine serum albumin protein was applied to saturate any excess protein-binding sites. The blocked sections were incubated with E-cadherin antibody (GTX100443, diluted 1:200, rabbit polyclonal), vimentin antibody (GTX112661, diluted 1:200, rabbit polyclonal), anti-HMGB1 (ab79823; diluted 1:350, rabbit monoclonal), and anti-PPAR-γ (Novusbio, diluted 1:100, rabbit polyclonal) at 4°C overnight. After the tissues had been rinsed three times with PBS for 5 min, a two-step immunohistochemical detection kit (G1210-2, Servicebio, Wuhan, China) was used. After incubation with horseradish peroxidase-conjugated secondary antibody and visualization with diaminobenzidine color according to the manufacturer's instructions, the slides were stained with hematoxylin, dehydrated in a graded ethanol series, and xylene transparent. Finally, the slides were observed under a light microscope.

### Immunofluorescence

For immunofluorescence histochemistry, the ECRSwNP and control tissues were fixed with 4% paraformaldehyde at 4°C overnight. After washing, they were gradient transferred into 20%, 30% sucrose in PBS for dehydration and cryoprotection. The tissues were embedded in OCT (Sakura, Tokyo, Japan) and cut into sections with a thickness of 10 μm using a cryostat (CM3050s, Leica, Germany). For immunofluorescence cytochemistry, the primary human nasal epithelial cells were incubated with or without rhHMGB1 or ROG in 24-well plates for 48 h. Then, the sections and cells were incubated with blocking solution (10% donkey serum with 0.2% Triton X-100) for 1 h at room temperature, then incubated with primary antibody (E-cadherin, vimentin, and HMGB1 for slides and ZO-1 and vimentin for cells) overnight at 4°C. After rinsing, the sections and cells were incubated with Alexa Fluor 594 Donkey Anti-Rabbit IgG (H+L), diluted 1:200, for 1 h, and counterstained with 4,6-diamidino-2-phenylindole (Roche, Shanghai, China) for the staining of nuclei. After washing with PBS, the cover slips were mounted with antifade mounting medium (Beyotime, Shanghai, China) on slides. Images were taken with a laser scanning confocal microscope (Nikon A1-si, Japan). The reconstruction images were produced with the NIS-Element software (Nikon, Japan).

### Western blotting

The protein expression level was examined using western blot. ECRSwNP tissues, control tissues, and incubated cells were dissected and lysed in radioimmunoprecipitation assay lysis solution (Beyotime, Haimen, Jiangsu, China) containing phosphatase inhibitors and PMSF. The protein concentrations were measured using the bicinchoninic acid protein assay kit (Beyotime Haimen, Jiangsu, China). 20μg protein was loaded on 10% SDS-polyacrylamide gel and it was separated. The proteins in the gel were then transferred to polyvinylidene difluoride membranes, which were incubated in blocking solution (5% non-fat milk in Tris-buffered saline) for 1 h at room temperature and then incubated overnight at 4°C with primary antibodies containing: ZO-1 (GTX108613, CA, USA, rabbit polyclonal), E-cadherin (GTX100443, CA, USA, rabbit polyclonal), N-cadherin (GTX127345, CA, USA, rabbit polyclonal), vimentin (GTX112661, CA, USA, rabbit polyclonal), in a dilution ratio of 1:1000, and HMGB1 (ab79823, Abcam, rabbit monoclonal), in a dilution ratio of 1:10,000. The membranes were washed three times with TBST and incubated with secondary antibodies, horseradish peroxidase-conjugated goat anti-rabbit or goat anti-mouse IgG (diluted 1:4000) for 60 min at room temperature. An electrochemiluminescence kit (Beyotime, Haimen, Jiangsu, China) was used to visualize the membranes. The relative expression level of proteins was represented in the ratio of the target protein and GAPDH using ImageJ 1.51J8 software (National Institutes of Health, USA).

### Statistical analyses

All data were analyzed with SPSS (version 17.0). The measured data were presented as the mean ± standard deviation. Quantitative data were analyzed using the unpaired Student's *t*-test for comparing two groups and one-way analysis of variance for multiple-variant analyses. A P value of less than 0.05 was considered to indicate a statistically significant difference.

## Results

### EMT markers, HMGB1 and PPAR-γ expression patterns in ECRSwNP and control tissues

**Figure 1:**
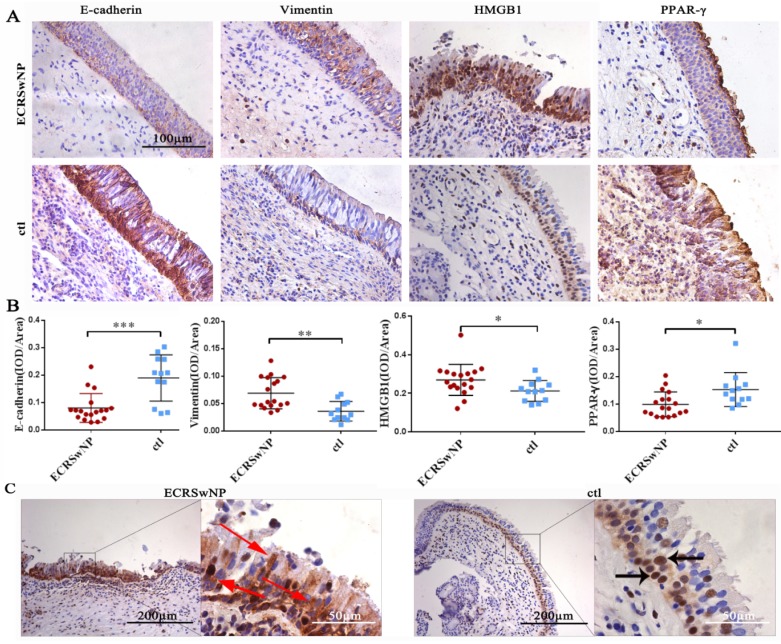
** Staining of E-cadherin, vimentin, HMGB1, and PPAR-γ in ECRSwNP and control tissues. (A)** Immunohistochemistry assay was performed to detect the staining of E-cadherin, vimentin, HMGB1, and PPAR-γ in ECRSwNP and control tissues. Bar, 100 μm. **(B)** The expression of E-cadherin, vimentin, HMGB1, and PPAR-γ in ECRSwNP and control tissues was determined by immunohistochemical semi-quantification. Data were represented as the mean ± SD (standard deviation). *P < 0.05, **P < 0.01, ***P < 0.001. ECRSwNP (*n* = 18), ctl (*n* = 12). **(C)** The location of HMGB1 in ECRSwNP and control tissues. Black bar, 200 μm, white bar, 50 μm. The red arrows showed the location of HMGB1 in the cytoplasm. The black arrows showed the location of HMGB1 in the nucleus.

**Figure 2:**
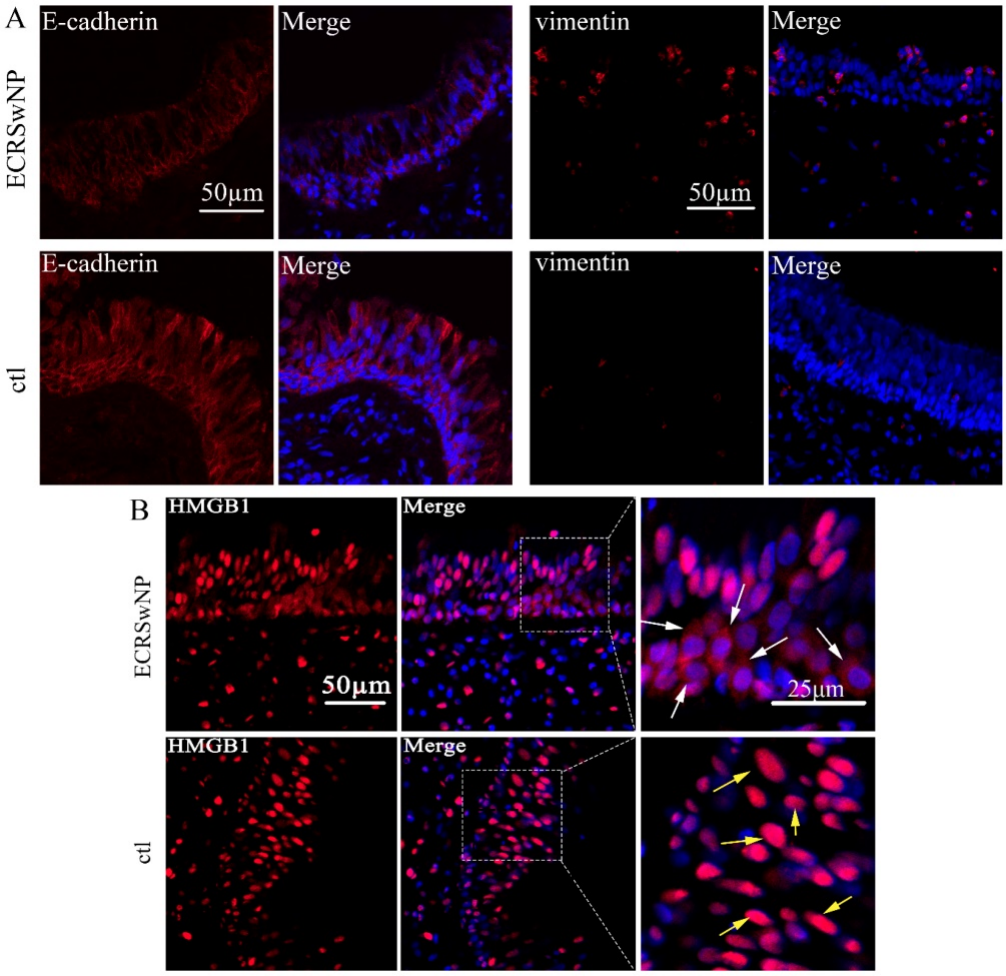
** Expression of E-cadherin, vimentin, and HMGB1 in ECRSwNP and control tissues. (A)** Confocal microscopic examination of E-cadherin and vimentin in ECRSwNP and control tissues. Bar, 50 μm. **(B)** Expression and location of HMGB1. The white arrows showed the location of HMGB1 in the cytoplasm. The yellow arrows showed the location of HMGB1 in the nucleus. Left bar, 50 μm, right bar, 25μm.

To evaluate the expression of EMT markers, HMGB1, and PPAR-γ in ECRSwNP, we performed IHC staining in 18 human ECRSwNP samples and 12 control tissues. IHC analysis of tissue sections revealed that the expression levels of E-cadherin and PPAR-γ were down-regulated in the ECRSwNP tissues, whereas those of vimentin and HMGB1 were up-regulated in the ECRSwNP tissues (Fig. 1A). Semiquantitative evaluation of IHC staining was used to detect the expression of E-cadherin, vimentin, HMGB1, and PPAR-γ in 18 ECRSwNP and 12 control tissue samples. There were significant differences in expression of E-cadherin (P < 0.001), vimentin (P < 0.01), HMGB1 (P < 0.05) and PPAR-γ (P < 0.05) (Fig. 1B). Obvious cytoplasmic staining of HMGB1 was observed in the ECRSwNP tissues compared with the control tissues (Fig. 1C). Confocal microscopy confirmed that the expression of E-cadherin was down-regulated, whereas vimentin was up-regulated in ECRSwNP (Fig. 2A). More cytoplasmic distribution of HMGB1 was found in ECRSwNP tissues (Fig. 2B).

### Expression of EMT markers in ECRSwNP and control tissues in western blots

**Figure 3:**
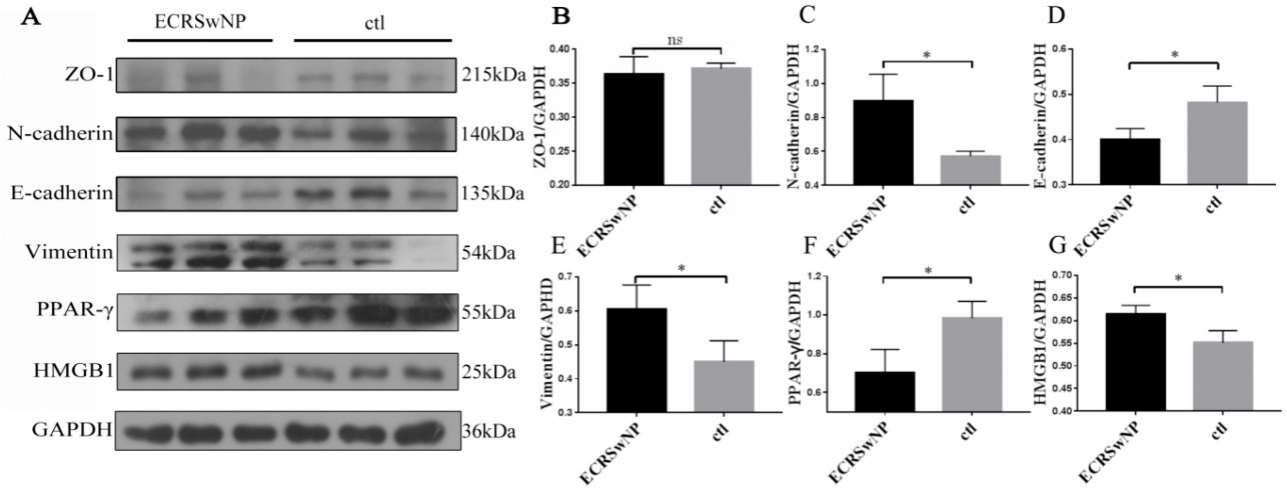
** Expression of EMT markers in ECRSwNP and ctl tissues. (A)** The protein expressions of ZO-1, N-cadherin, E-cadherin, vimentin, PPAR-γ and HMGB1 were examined by western blot. **(B, C, D, E, F, G)** Quantitative analyses of EMT markers, PPAR-γ and HMGB1. Data were expressed as mean ± SD. *P < 0.05.

The epithelial markers ZO-1 and E-cadherin were down-regulated in ECRSwNP compared with control, whereas the mesenchymal markers N-cadherin and vimentin were up-regulated in ECRSwNP compared with control. Expression of HMGB1 was obvious in ECRSwNP while expression of PPAR-γ was obvious in control tissues (Fig. 3A). Quantitative analyses of protein expression levels of N-cadherin, E-cadherin, vimentin, PPAR-γ and HMGB1 in the western blot was significantly different (P < 0.05) (Fig. 3C, D, E, F, G). Visual observation of the band confirmed that the expression of ZO-1 was lower in ECRSwNP tissues, but there was no significant statistical difference (ZO-1, P = 0.1048) (Fig. 3B).

### RhHMGB1 promoted EMT in ECRSwNP cells

**Figure 4:**
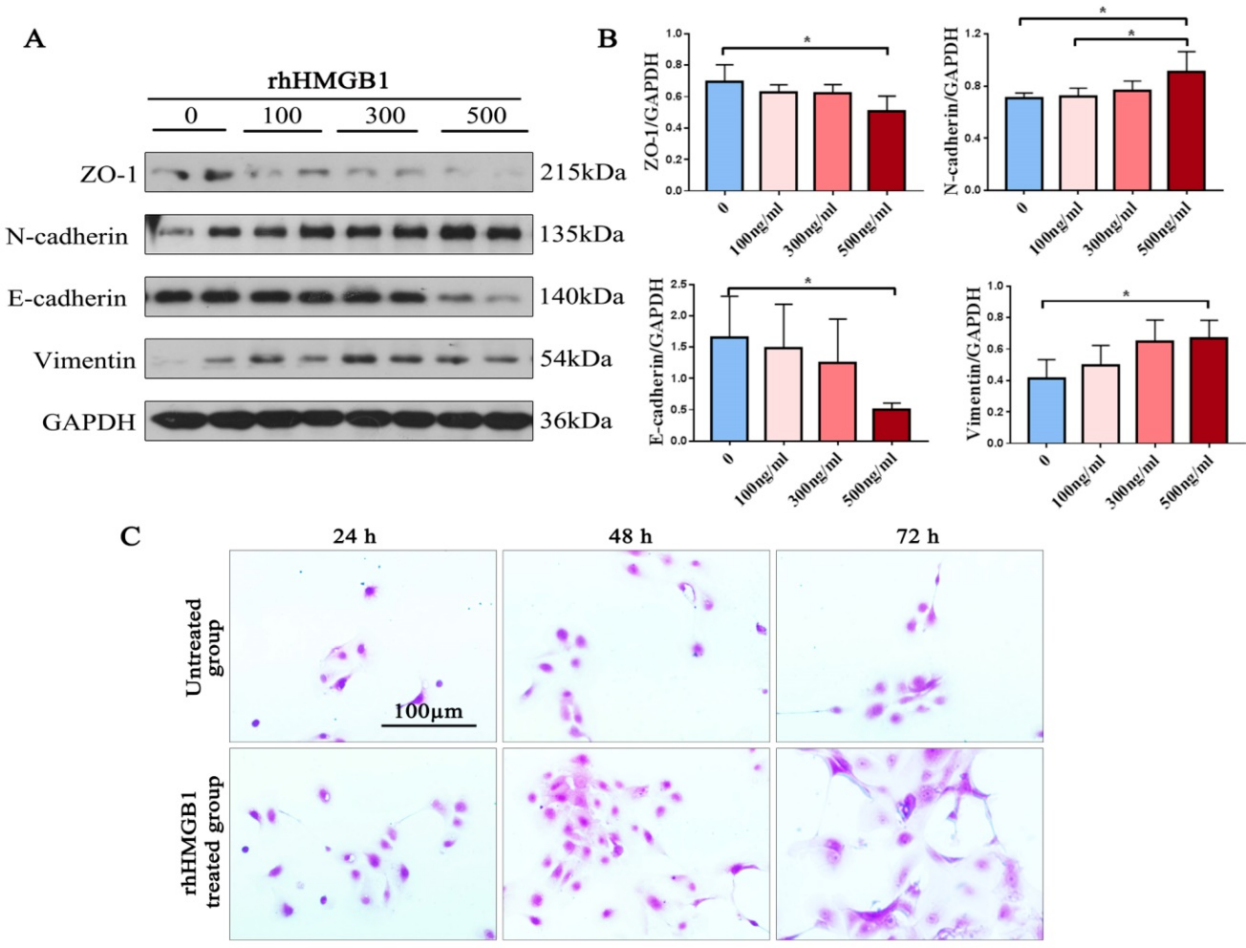
** Expression of EMT markers in preliminary NP cells. (A)** Western blot assay was performed to detect the expression of ZO-1, N-cadherin, E-cadherin, vimentin and GAPDH in primary ECRSwNP cells after treatment with dose-dependent rhHMGB1(ng/ml). **(B)** Quantitative analyses of expression levels of EMT markers in primary ECRSwNP cells. Data were expressed as mean ± SD. *P < 0.05. **(C)** Cell morphology in the induction group (rhHMGB1: 500ng/ml) and the control group at 24, 48, and 72 h. Cells were stained with crystal violet. Bar, 100 μm.

EMT markers were detected in rhHMGB1-incubated primary human nasal epithelial cells from ECRSwNP tissues via western blot. We found that rhHMGB1 can significantly decrease the epithelial markers E-cadherin and ZO-1, and increase the mesenchymal markers N-cadherin and vimentin in ECRSwNP cells in a dose-dependent fashion (Fig. 4A). RhHMGB1 (500ng/ml) induced Changes in the expression of EMT-related proteins and there was significantly different (P < 0.05). Additionally, cells incubated with rhHMGB1 underwent obvious morphological changes in comparison with the control group. The number of cells and the length of the pseudopodia increased in direct proportion to increased incubation time. This change may confer upon the cells a greater ability to migrate (Fig. 4C).

### Rosiglitazone inhibited rhHMGB1-induced EMT

**Figure 5:**
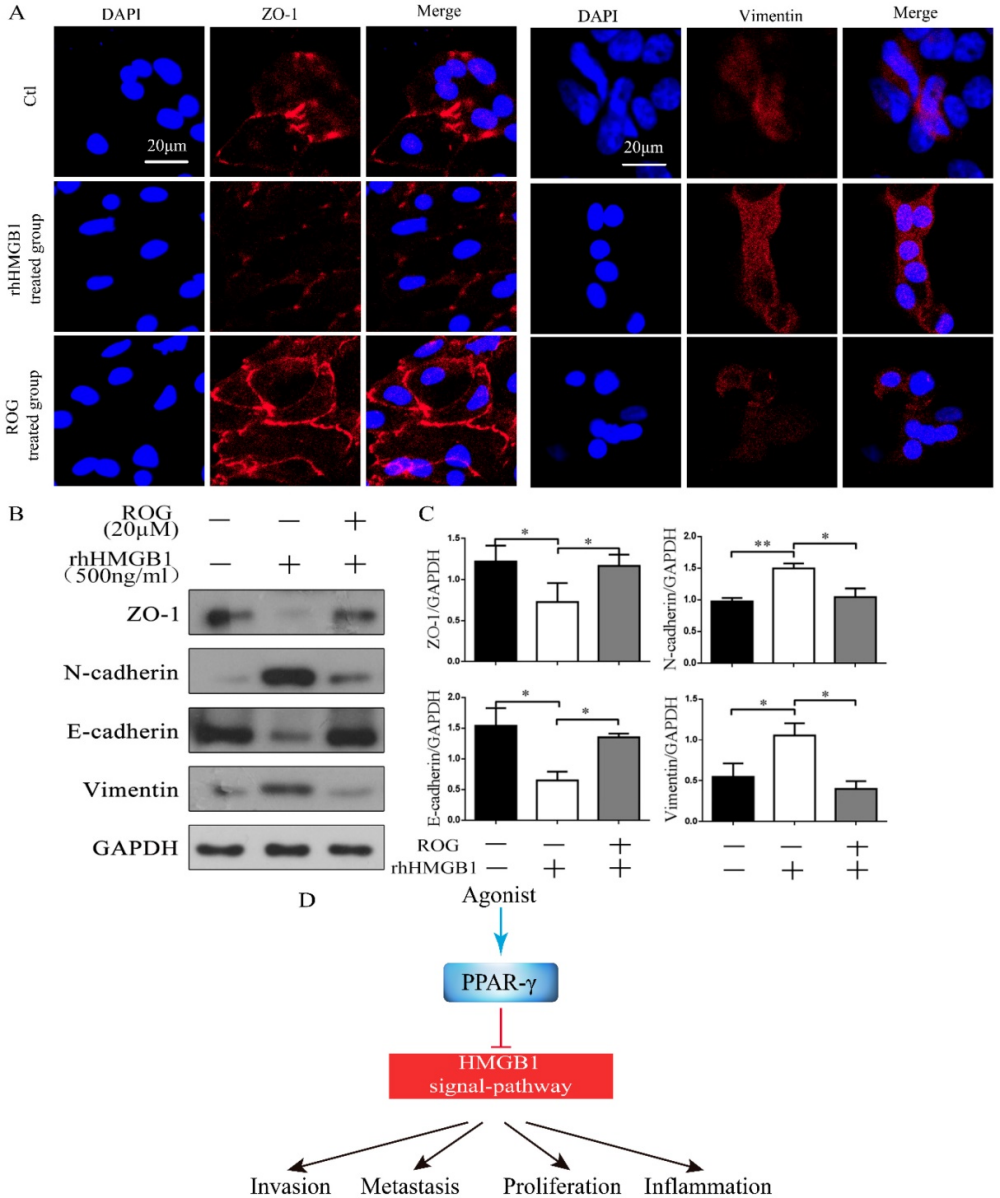
** ROG inhibited rhHMGB1-induced EMT. (A)** Confocal microscopic examination of ZO-1 and vimentin in different groups of ECRSwNP cells. Scale bar, 20 μm. **(B)** Effect of ROG (20µM) on the changes of EMT markers expression in ECRSwNP cells. Cells were cultured with or without rhHMGB1 (500ng/ml). **(C)** Quantitative analyses of ZO-1, N-cadherin, E-cadherin and vimentin. Data were expressed as mean ± SD. *P < 0.05 **P< 0.01. (D) Agonist of PPAR-γ inhibited HMGB1-induced EMT.

After incubation with rhHMGB1 for 48 h, the expression of the intercellular adhesion molecule ZO-1 was decreased and vimentin was increased, whereas the addition of ROG (20 µM) after incubation with rhHMGB1 (500 ng/ml) for 30 min enhanced the expression of ZO-1 but weaken expression of vimentin in confocal microscopic examination (Fig. 5A). RhHMGB1 down-regulated the expression of ZO-1 and E-cadherin and up-regulated the expression of N-cadherin and vimentin, while the agonist of PPAR-γ, ROG restored this change (Fig. 5B). The inhibitory effect of ROG on rhHMGB1-induced EMT was statistically significant (Fig. 5C).

### ROG inhibited LPS induced generation of HMGB1 and suppressed the following EMT

**Figure 6:**
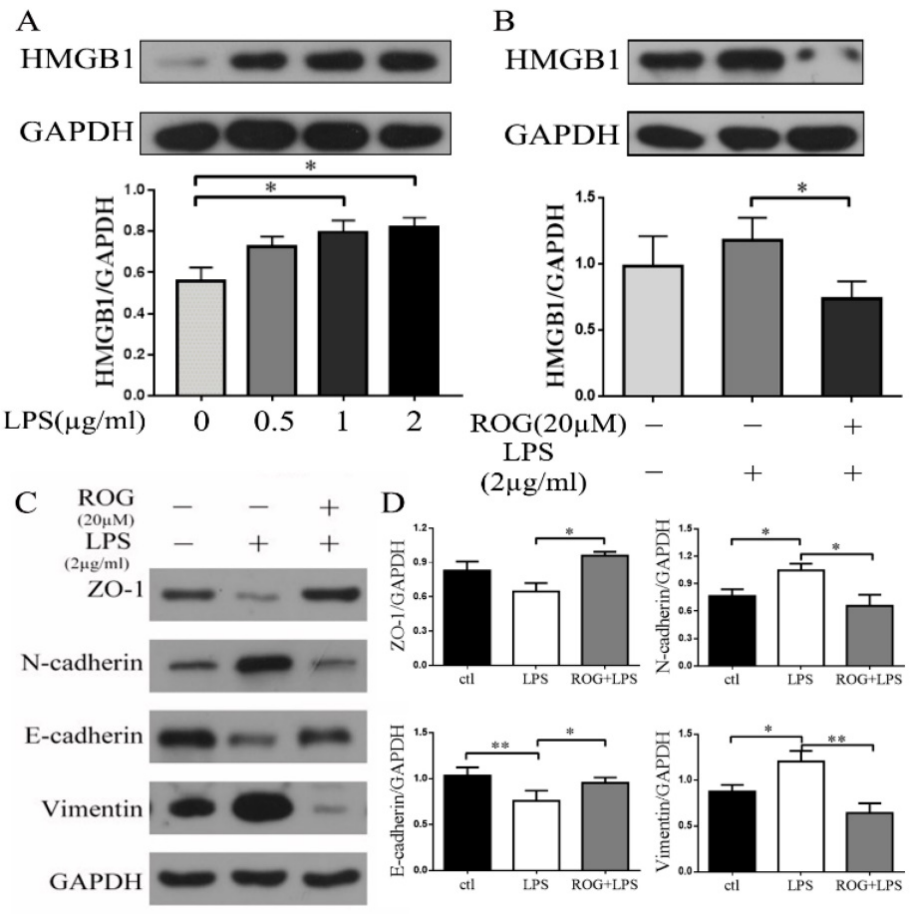
** ROG inhibited LPS-induced EMT via HMGB1. (A)** Effect of dose-dependent LPS on the expression of HMGB1. Cells were cultured with or without LPS. **(B)** Effect of ROG on the changes in HMGB1 expression with LPS. **(C)** Effect of ROG on the changes of EMT markers expression in ECRSwNP cells. Cells were cultured with or without LPS. **(D)** Quantitative analyses of ZO-1, N-cadherin, E-cadherin and vimentin. Data were expressed as mean ± SD. *P < 0.05 **P< 0.01.

LPS increased HMGB1 expression in a dose-dependent manner. Both 1µg/ml and 2µg/ml can significantly promote the expression of HMGB1 and there was significant different ((P < 0.05) (Fig. 6A). LPS (2µg/ml) induced the generation of HMGB1, while the agonist of PPAR-γ (ROG, 20 µM) reversed the expression of HMGB1 (Fig. 6B). Incubated ECRSwNP cells with LPS (2µg/ml) induced down-regulated expression of ZO-1 and E-cadherin and up-regulated expression of vimentin and N-cadherin. ROG restored this change (Fig. 6C). The difference of EMT markers expression was statistically significant after intervention with LPS (Fig. 6D).

## Discussion

Inflammation and tissue remodeling are major morbidity factors in CRSwNP and there are some links between them. According to Hellquist's classification, the histological hallmarks of ECRSwNP contained edematous stroma, hyperplasia goblet cells, thickened basement membrane and numerous eosinophil cells while nonECRSwNP instead of more fibrosis tissues and infiltrate more lymphocyte cells 30. There are positive correlations between edema and eosinophilic inflammation and between fibrosis and neutrophilic inflammation in CRSwNP 31-33. So clinically, ECRSwNP appears friable, readily disintegrate, whereas nonECRSwNP is not edematous and instead of density tissues and pathologically, nonECRSwNP is distinguished by vigorous glandular hypertrophy compared with ECRSwNP 33,34. About the mechanism of tissue restructuring, EMT plays an important role. EMT, or transformation from epithelial to mesenchymal states, which functions in embryonic development, inflammation, wound healing, and carcinoma, has also been reported to be involved in the tissue remodeling of CRSwNP 35,36.

Many factors through different pathway regulate EMT, such as TGF-β1/SMAD, PI3K/AKT, MARK/ERK, and Wnt/β-catenin signaling pathway to achieve the purpose of organizing tissue remodeling. Although TGF-β1 was the most active player in pathogenesis of EMT, it was belongs to Th1 and Th3 cytokines and its expression in ECRSwNP was not obvious compared with non-ECRSwNP 37. In previous studies, the expression trend of TGF-β1 in CRSsNP, non-ECRSwNP and ECRSwNP is gradually declining 38-40. And that, the EMT in ECRSwNP was more pronounced than in non-ECRSwNP. There must be other related factors causing the occurrence of EMT in ECRSwNP. In exploring the possible factors of inducing inflammation and tissue remodeling of ECRSwNP, which has the characteristics of abundant eosinophil infiltration, we found that the survival, migration, adhesion, and degranulation of eosinophils was depend to a great extent on HMGB1 41. Furthermore, varying expression patterns of HMGB1 have been observed in patients with asthma, COPD, endometrial carcinoma, bladder cancer, and lung cancer, and the pathogenesis of all has been considered to be importantly related to EMT 42-44. In present study, HMGB1 was up-regulated in ECRSwNP tissues compared with control tissues. It's Consistent with previous studies. For the expression of HMGB1 in ECRSwNP was the highest among ECRSwNP, non-ECRSwNP, CRSsNP and normal tissues in previous studies, we thought HMGB1 has an important effect on the occurrence of EMT in ECRSwNP 41,45. Moreover, we investigated the location of HMGB1. Our IHC assay revealed that cytoplasmic staining of HMGB1 was pronounced in the ECRSwNP tissues compared with control tissues. This result is supported by previous research in which the pro-inflammatory function of extracellular HMGB1 was elucidated 46.

HMGB1, a non-histone nuclear protein, regulates a number of key DNA events, such as nucleosome stability and sliding, genome chromatinization, DNA binding, DNA bending, and repairs 47-49. Schaefer et al. proved that HMGB1 induces EMT through the RAGE and the PI3K/AKT/GSK3β/β-catenin signaling pathway in human airway epithelial cells 50. In the process of EMT, the expression of epithelial markers decreases, whereas the expression of mesenchymal markers increases. In our study, the expression of mesenchymal markers such as N-cadherin and vimentin increased, and the expression of E-cadherin and ZO-1 decreased in ECRSwNP tissues compared with the control tissues. The evidence indicated the occurrence of EMT in ECRSwNP. In view of the positive regulation between HMGB1 and eosinophils 51, and the role of HMGB1 in causing EMT, we believe that EMT is partially induced by HMGB1 in ECRSwNP and that the inhibition of HMGB1 expression may help to inhibit the EMT process. In present study, we found that the presence of EMT in ECRSwNP was accompanied by an increase of HMGB1 expression obviously. As an *in vitro* test to confirm the role of HMGB1 in ECRSwNP, we treated cells which were isolated from ECRSwNP tissues with rhHMGB1. We found that rhHMGB1 induced EMT dose-dependently. Furthermore, rhHMGB1 treatment induced morphological changes in ECRSwNP cells. Confocal microscopic assay also confirmed the function of HMGB1-induced EMT.

PPAR-γ, which belongs to a nuclear hormone receptor superfamily, has the function of modulating lipid/lipoprotein metabolism, cell cycle progression, cellular proliferation, and differentiation after binding to ligand 52,53. It was reportedly has an anti-inflammatory function and has potential value in the treatment of COPD, idiopathic pulmonary fibrosis, asthma, acute lung injury and acute respiratory distress syndrome 29,54-56. Previous research has indicated multiple effects of PPAR ligands, such as telmisartan, troglitazone, rosiglitazone, and pioglitazone, suppressing the biological actions of HMGB1 57-59. Rosiglitazone, a member of the thiazolidinedione class, has been reported to have a suppressive effect on LPS-induced acute injury via inhibition of HMGB1 through RAGE/NF-κB and MAPK pathways 60. In order to explore the effect of ROG on exogenous rhHMGB1, we used ROG to treat the cells which were interfering with rhHMGB1. We found that ROG administration reversed the effect of rhHMGB1 on EMT in ECRSwNP cells. To explore the effect of ROG on endogenous HMGB1, we used LPS to stimulate the expression of HMGB1. Many previous literatures support the release of HMGB1 and the subsequent release of inflammatory mediators were stimulated by LPS 61,62. Consistent with previous studies, we found LPS increased expression of HMGB1 combined with amplification of EMT. Incubating cells with ROG, the results showed that ROG inhibited LPS-induced HMGB1 release and followed the inhibition of EMT in cells isolated from ECRSwNP tissues. These results demonstrate that the agonist of PPAR-γ has the same effect on extracellular HMGB1 and endogenous HMGB1. It can inhibit the biological action of HMGB1 at different levels.

The limitation of the present study is that the sample size of ECRSwNP patients and control subjects is small. In addition, this finding was specific on ECRSwNP, which demonstrate the effect of HMGB1 on pathogenesis of ECRSwNP, but was not differentiated into ECRSwNP, non-ECRSwNP, CRSsNP and normal tissues, which may illustrate the difference of HMGB1 in the pathogenesis of chronic inflammatory disease in the nasal cavity. Thus, further studies involving larger groups of patients and classified into different endotypes are needed in the future.

In summary, our research found high expression of HMGB1 in ECRSwNP tissues and showed that HMGB1 can induce EMT in ECRSwNP cells partially. The agonist of PPAR-γ inhibits HMGB1-induced EMT procession. The ligand of PPAR-γ may be a potential agent for the treatment of ECRSwNP.

## Figures and Tables

**Figure 7:**
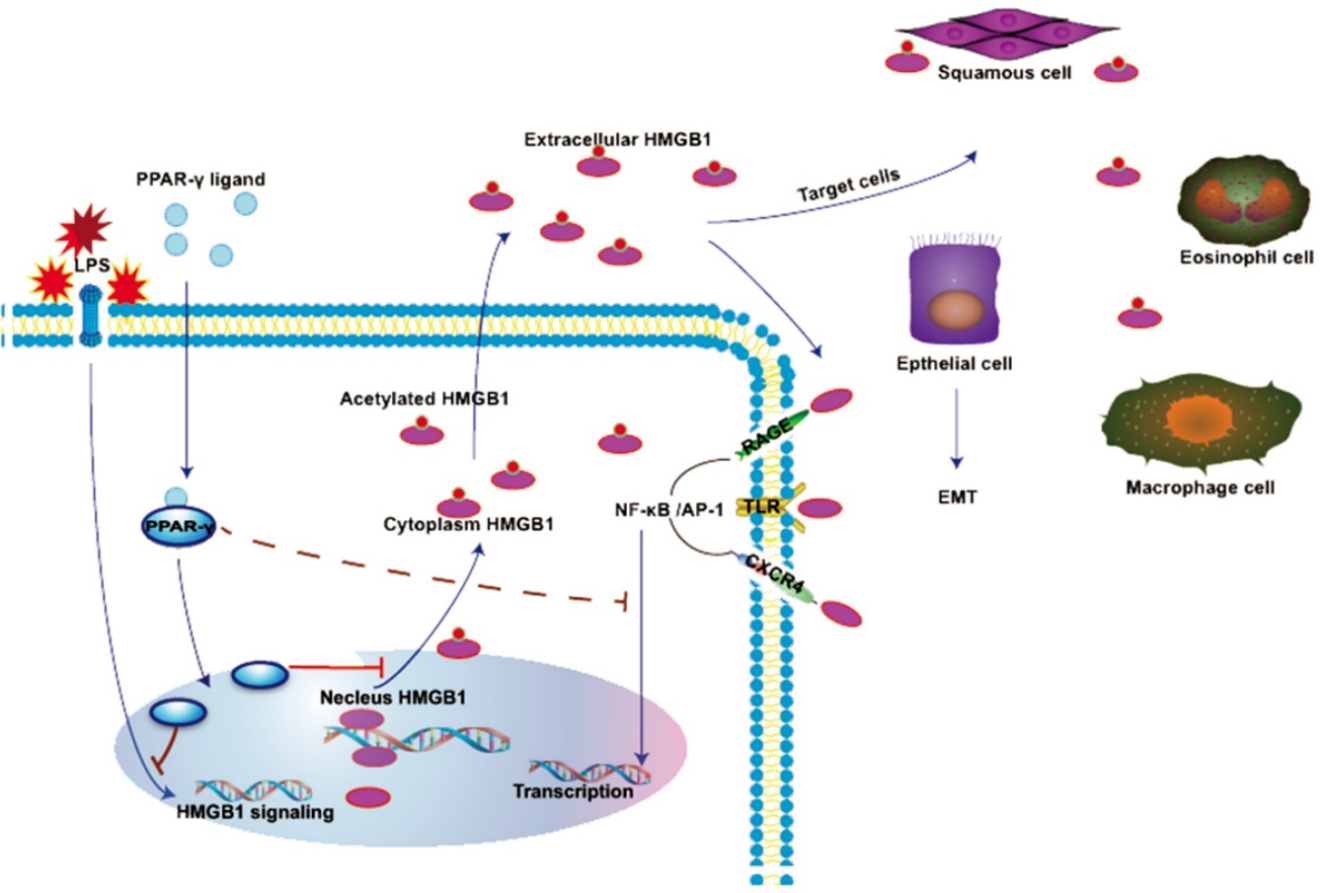
**In normal conditions, HMGB1 is mainly localized in the nucleus in a non-acetylated form.** HMGB1 binds RAGE, TLRs, or CXCR4 to induce NF-кB activation. NF-кB translocate to the nucleus and cause EMT in several cells. HMGB1 is actively secreted by LPS-stimulated cells and translocated to the cytoplasm or extracellular space. The ligand of PPAR-γ inhibited generation and translocation of HMGB1 stimulated by LPS, whereas the effect of rhHMGB1 is also inhibited by the agonist of PPAR-γ.
